# Molecular Changes in *Opisthorchis viverrini* (Southeast Asian Liver Fluke) during the Transition from the Juvenile to the Adult Stage

**DOI:** 10.1371/journal.pntd.0001916

**Published:** 2012-11-29

**Authors:** Aaron R. Jex, Neil D. Young, Jittiyawadee Sripa, Ross S. Hall, Jean-Pierre Scheerlinck, Thewarach Laha, Banchob Sripa, Robin B. Gasser

**Affiliations:** 1 Faculty of Veterinary Science, The University of Melbourne, Parkville, Victoria, Australia; 2 College of Medicine and Public Health, Ubon Ratchathani University, Ubon Ratchathani, Thailand; 3 Department of Parasitology, and Liver Fluke and Cholangiocarcinoma Research Center, Faculty of Medicine, Khon Kaen University, Khon Kaen, Thailand; 4 Tropical Disease Research Laboratory, Department of Pathology, Faculty of Medicine, Khon Kaen University, Khon Kaen, Thailand; University of Queensland, Australia

## Abstract

**Background:**

The Southeast Asian liver fluke (*Opisthorchis viverrini*) chronically infects and affects tens of millions of people in regions of Asia, leading to chronic illness and, importantly, inducing malignant cancer ( = cholangiocarcinoma). In spite of this, little is known, at the molecular level, about the parasite itself, its interplay with its hosts or the mechanisms of disease and/or carcinogenesis.

**Methodology/Principal Findings:**

Here, we generated extensive RNA-Seq data (Illumina) representing adult and juvenile stages of *O. viverrini*, and combined these sequences with previously published transcriptomic data (454 technology) for this species, yielding a combined assembly of significantly increased quality and allowing quantitative assessment of transcription in the juvenile and adult stage.

**Conclusions:**

This enhanced assembly reveals that, despite the substantial biological similarities between the human liver flukes, *O. viverinni* and *Clonorchis sinensis*, there are previously unrecognized differences in major aspects of their molecular biology. Most notable are differences among the C13 and cathepsin L-like cysteine peptidases, which play key roles in tissue migration, immune evasion and feeding, and, thus, represent potential drug and/or vaccine targets. Furthermore, these data indicate that major lineages of cysteine peptidases of socioeconomically important trematodes have evolved through a process of gene loss rather than independent radiation, contrasting previous proposals.

## Author Summary

*Opistorchis viverrini* is an important and neglected parasite affecting ∼9 million people in South-east Asia. The parasite has a complex life-cycle which involves an intermediate phase in cyprinoid fishes. Consumption of raw or under-cooked fish infected with the metacercarial (larval) stage of *O. viverreni* results in infection, with adult worms living primarily in the intra-hepatic bile duct. In addition to the affects of the infection itself, *O. viverrini* is directly carcinogenic, with up to 70% of infected individuals in endemic regions developing malignant cholangiocarcinomas. Control of the parasite relies exclusively on the use of praziquantel and little is known about the mechanisms through which *O. viverrini* stimulates carcinogenesis. An improved understanding of the molecular biology of *O. viverrini* is urgently needed. In our study, we employed RNAseq technology to assess changes in gene transcription during the development of *O. viverrini* within the definitive host, and significantly improved the characterization of the transcriptome of this parasite. In so doing, we shed new light on the evolution of a major group proteins (i.e., the cysteine peptidases) which, given their important function roles as excreted/secreted molecules, have been proposed as attractive drug/vaccine targets for a wide-range of neglected flukes, including species of *Opistorchis*, *Clonorchis*, *Schistosoma* and *Fasciola*.

## Introduction

Parasitic worms of humans and other animals cause diseases of major socio-economic importance around the world. In spite of their significance, many of them have been substantially neglected in terms of research and their control [1]. Among parasitic flukes (i.e., trematodes), the foodborne trematodes, including the major human liver flukes, *Opisthorchis viverrini* and *Clonorchis sinensis*, are particularly understudied. In parts of Southeast Asia, including Cambodia, People's Democratic Republic of Laos, Thailand and Vietnam, *O. viverrini* is estimated to infect ∼9 million people [2], with ∼67 million being at risk of infection [3].

The life-cycle of this parasite is complex, involving multiple intermediate hosts and a prey-to-predator transmission cycle [4]. Briefly, embryonated eggs are shed into the environment in the faeces from the infected definitive host (mainly humans, dogs and cats). After the eggs are shed into water (usually *via* untreated sewage), they are ingested by freshwater snails (*Bithynia* spp.) and then hatch in the gut, releasing the motile embryo ( = miracidium), which develops into a sporocyst. Asexual reproduction within the sporocyst gives rise to rediae and then cercariae. The motile cercariae are released from the snail into the aquatic environment. Thereafter, these larvae undergo host finding and must penetrate the skin of a cyprinoid fishes (e.g., *Puntius* spp.) and encyst as metacercariae within the skin and/or musculature to survive. The piscivorous definitive hosts become infected upon ingestion of such fish in a raw or undercooked state [5]. Following gastric passage, the metacerariae excyst in the duodenum, and the juvenile flukes migrate *via* the ampulla of Vater and common bile duct to the intra-hepatic bile and/or sometimes into the pancreatic ducts (∼14 days), whereupon they develop into reproductively-active, hermaphroditic adults (∼4 weeks), which release embryonated eggs via bile/pancreatic fluid into chyme and then *via* host faeces into the freshwater environment, thus completing the life cycle [4].

Although infection is often asymptomatic, signs or symptoms associated with opisthorchiasis can include eosinophilia and, in intense infections, diarrhoea, epigastric pain, anorexia, pyrexia, jaundice and/or ascites [6]. Chronic opisthorchiasis often leads to cholangitis, periductal fibrosis, cholecystitis and/or cholelithiasis, and, in up to 71% of infected humans in endemic areas, can induce malignant cancer (cholangiocarcinoma) [7]. Hence, *O. viverrini* has been classified as a Group I carcinogen [8]. In endemic regions, sanitation infrastructure is often limited, and cyprinoid fish is consumed in a variety of traditional dishes. For cultural reasons, this fish is often eaten raw, and there is a resistance to recommendations to cook fish to prevent the transmission of opisthorchiasis. Therefore, the only practical measure to reduce the prevalence of cholangiocarcinoma is the treatment of *O. viverrini* infection with praziquantel [5]. However, the reliance on this sole treatment carries a significant risk that drug resistance will develop against this compound, as has been observed for trematocidal drugs in other flukes [9].

Clearly, understanding the intricacies of the biology of *O. viverrini* and opisthorchiasis is central to developing new and urgently needed intervention strategies. Yet, in spite of our knowledge of the morphological changes that occur in the parasite throughout its life cycle and its paramount importance as a carcinogen, we know very little about the molecular basis of the developmental biology of *O. viverrini*, its interactions with its hosts and the pathogenesis of disease, particularly carcinogenesis. The advent of next-generation sequencing and bioinformatic technologies [10], [11] now provides unprecedented opportunities to address some of these key areas. Recently, Young and coworkers [12] characterised the transcriptome of *O. viverrini* using 454 sequencing technology, which provided a solid basis to explore, for the first time, the transcriptional profiles of different developmental stages of this parasite. Logically extending this work, we now characterize differential transcription between adult and juvenile stages of *O. viverrini* using the method of RNA-Seq (Illumina technology) [13], and identify key molecules inferred to be associated with development, reproduction, feeding and survival of this neglected parasite.

## Materials and Methods

### Ethics statement

Hamsters used in this study were maintained at the animal research facilities at the Faculty of Medicine, Khon Kaen University, Thailand. All work was conducted in accordance with protocols approved by the Animal Ethics Committee of Khon Kaen University (reference number #0514.1.12.2/23) in accordance with the ‘Animal for Scientific Purposes Act’ of the National Research Council of Thailand.

### Collection and identification of *O. viverrini*

Using established methods [14], metacercariae were collected from infected cyprinoid fishes from the Khon Kaen province of Thailand and used to orally infect eight helminth-free, inbred Syrian (golden) hamsters (*Mesocricetus auratus*) in Khon Kaen University. Hamsters were euthanized 14 and 42 days following inoculation with metacercariae, in order to collect juvenile and adult stages of *O. viverrini*, respectively. Worms (all stages) were expressed from the intra- and extra-hepatic bile ducts and cultured *in vitro* for 2 h, to stimulate the regurgitation of their caecal contents [12] prior to being washed extensively in physiological saline (25°C), snap-frozen in liquid nitrogen and then stored at -80°C. The specific identity of the flukes was verified by first isolating genomic DNA [15] and then carrying out PCR-coupled, bidirectional sequencing (ABI 3730×l DNA analyzer, Applied Biosystems, California, USA) of the second internal transcribed spacer (ITS-2) of the nuclear ribosomal DNA [16]. These sequence data were compared by pairwise alignment to published sequence data in the National Center for Biotechnology Information (NCBI) GenBank archive (accessible via www.ncbi.nlm.nih.gov:AY584735).

### Purification of mRNA and Illumina sequencing

Total RNA was isolated each from pools of adult (n = 15) or juvenile (n = 40) *O. viverrini* using the TriPure (Roche) reagent [17] and treated with DN*ase* I (TurboDNA-free, Ambion) according to the manufacturer's instructions. In order to control for variation in transcription due to host-related factors (e.g., immunological/genetic differences), these pools were constructed from individuals isolated from worms collected from each of the infected host animals. Total RNA concentrations were estimated spectrophotometrically, and RNA integrity was verified by agarose gel electrophoresis and using a 2100 BioAnalyzer (Agilent). Polyadenylated (polyA+) RNA was purified from 10 µg of total RNA using Sera-Mag oligo(dT) beads, fragmented to a length of 100–500 nucleotides, reverse-transcribed using random hexamers, end-repaired and adaptor-ligated, according to a recommended protocol (Illumina). Ligated products of 200 bp were excised from an agarose gel, PCR-amplified for 15 cycles, purified over MinElute column (Qiagen) and sequenced (single-end) on a Genome Analyzer II (Illumina).

### Combined 454 and Illumina assembly, and annotation

Following sequencing, the quality of all RNA-Seq data was assessed; only sequences with a PHRED score of ≥30 and a length of ≥40 nucleotides (nt) were retained. RNA-Seq data from the adult and juvenile libraries were combined with 454 data generated from adult *O. viverrini* in a previous study [12] and assembled using the program OASES v.0.1.21 [18]. The *k*-mer and coverage cut-off were optimized to achieve the best assembly with the greatest mean length of contiguous sequences and the fewest incomplete transcripts. Reads predicted to represent mitochondrial, ribosomal, host or microbial sequences, based on a BLASTn comparisons with sequences in the NCBI non-redundant database (accessible *via* www.ncbi.nlh.nih.gov), were removed from the dataset prior to subsequent analysis. Each transcript was conceptually translated in six frames, using customized PERL scripts, with the longest opening reading frame (ORF) being used to define the coding domain and its inferred amino acid sequence.

Following data assembly, the combined 454/Illumina transcripts were subjected to analysis/annotation using an established semi-automated, bioinformatic pipeline [12], [19] and compared (by BLASTx analysis at an *E*-value cut-off of <10^−05^, 10^−15^ and 10^−30^) with conceptually translated proteins from previously published transcriptomic data for *O. viverrini* [12], *C. sinensis* [12], *Fasciola* spp. [19], [20], as well as coding domains predicted from the genomes of *Schistosoma* spp. [17], [21], [22]. Functional annotation of each predicted peptide was inferred using multiple methods, including BLASTx comparison (cut-off: *E*-value: <10^−5^) with the non-redundant sequence database (March, 2012) available *via* GenBank (NCBI; http://www.ncbi.nlm.nih.gov/est/) and the UniProt database [23], prediction of conserved protein domains using InterProScan [24], allowing assignments of parental gene ontology (GO) terms (http://www.geneontology.org/) and homology-based mapping to conserved biochemical pathways in the Kyoto Encyclopaedia of Genes and Genomes (KEGG) using the KEGG orthology-based annotation system (KOBAS) [25]. Excretory/secretory (ES) proteins were predicted on the basis of the presence of a signal peptide at the N-terminus and absence of a transmembrane domain using the program PHOBIUS [26] and/or the identification of close homologues (BLASTp analysis: E-value cutoff <10^−05^) in the signal peptide database (SPD) [27] or a custom-built ES database containing published proteomic data for nematodes [28] and trematodes [17]. Peptidases and their inhibitors were predicted by BLASTx comparison with the MEROPS database (January 2011) [29]. The additional annotation of specific, key protein classes was achieved by BLASTx comparison with the KS-Sarfari and GPCR-Sarfari (http://www.sarfari.org) and the Transporter Classification (TCDB) [30] databases.

### Comparison of transcriptomes

The transcriptome assembled here using both 454 and Illumina data was compared qualitatively and quantitatively with the 454-based transcriptome published previously for the adult stage of this species [12]. Both assemblies were compared based on common assembly metrics (i.e., number of contigs, N50 and N90 metrics [28], largest contigs, number of annotated proteins, mean predicted peptide length and largest predicted peptide) using established bioinformatic approaches [28]. One-to-one orthologous transcripts (transcript = a ‘true’ mRNA represented by the contigs constructed during the assembly of the 454 and/or 454+ Illumina data) were identified in the ‘old’ (i.e., 454) and ‘new’ (i.e., 454+ Illumina) assemblies using the reciprocal best-hit method [31] based on the BLASTn algorithm. Using this approach, we identified transcripts common to both datasets and unique to each, and then assessed the functional annotation data available for each of these transcript groups (i.e., common to both, unique to the ‘old’ assembly and novel to the ‘new’ assembly). Where possible, we attempted to assess the support for these BLASTn comparisons by mapping all Illumina reads to the contigs representing these sequences using the Burrows-Wheeler Aligner (BWA) program [32].

Because homopolymer errors (i.e., indels) represent a known limitation of 454 sequencing [33], and had been predicted in the previously published *O. viverrini* transcriptome [12], we explored the extent to which the addition of Illumina RNA-Seq data included here was able to correct these errors and the frequency with which such repairs improved/restored the predicted ORF of each contig. To do this, we conducted reciprocal pairwise Smith Waterman alignments of each contig in each assembly using the BWA-SW command in the BWA program [32]. These pairwise alignments were filtered for the best (i.e., most similar) alignment for each contig pair, and interrogated for insertions or deletions associated with homopolymers of ≥4 nucleotides (i.e., indels in the 454 only assembly that were corrected through the addition of the Illumina data).

### Analysis of transcription

Following the optimization of the ‘new’ transcriptome for *O. viverrini*, we explored differential transcription between the adult and juvenile stages of this species using the RNA-Seq data for each stage. To account for large differences in the numbers of reads generated for these two libraries [34], we randomly sub-selected sequence reads (n = 7,527,263) from each library and aligned ‘adult’ and ‘juvenile’ read pools to the final transcriptome using the program SOAP2 [32], requiring that each read mapped exclusively to one location in the transcriptome with a minimum alignment length of 40 nt and a maximum of three nucleotide mismatches per read. Relative levels of transcription in adult and juvenile stages were inferred based on the calculation of reads per kilobase per million mapped reads (RPKM) [35]; statistical differences in quantitative transcription between the juvenile and adult stages of *O. viverrini* was determined using a modified Audic-Claire equation [36] relating to a Bonferroni transformed p-value (i.e., False Discovery Rate) of ≤0.01 and ≥2-fold absolute difference in RPKM levels, as described previously [28].

### Phylogenetic analysis

To assess the expansion of the cysteine peptide domain proteins in *O. viverrini* relative to the other parasitic trematodes for which extensive transcriptomic/genomic data are available, we compiled all representative sequences in the present optimized transcriptome or available from transcriptomic data for *C. sinensis* [12] and *Fasciola* spp. [19], [20] as well as genomic data for *Schistosoma* spp. [17], [21], [22]. We extracted the nucleotide region encoding the C13 legumain-like (PF01650) or C1 cathepsin-like (PF01650) cysteine peptidase domains from each of these sequences, aligned these data using the program MUSCLE [37] through 50 iterations and manually verified this alignment by visual inspection in BioEdit (http://www.mbio.ncsu.edu/bioedit/). To eliminate redundancy, a complete or nearly complete transcript for each unique C13 legumain-like or C1 cathepsin-like domain detected in each alignment was retained as a representative of all sequences ( = contigs or singletons) assembled in these datasets. To assess evolutionary relationships between and among the C13 legumain-like or C1 cathepsin-like peptidases represented in this consensus alignment, phylograms were constructed by Bayesian inference (BI) using the program Mr Bayes v. 3.1.2 [38] employing the Monte Carlo Markov chain method (nchains = 4) over 1,000,000 tree-building generations, with every 100^th^ tree being saved; 10% of the saved trees were discarded (burnin = 1,000 trees) to ensure stabilisation of the nodal split frequencies, and consensus trees for each peptidase family were constructed from all remaining trees, with the nodal support for each clade expressed as a posterior probability (pp). The consensus trees were generated and labelled in Figtree (http://tree.bio.ed.ac.uk/software/figtree/).

## Results

We used an RNA-Seq-based approach to improve the transcriptome of *O. viverrini*, and to explore differential transcription between the juvenile and adult stages of this parasite. Following sequencing and quality filtering (PHRED quality ≥Q30), we generated 14,862,797 and 7,527,263 single-end sequence reads (mean length: 49 bp) from juvenile and adult *O. viverrini*, respectively and deposited raw data under the accession number SRA052929 in the sequence read archive of NCBI (http://www.ncbi.nlm.nih.gov/sra). This RNA-Seq data was then combined with 642,918 454-based sequence reads (mean length: 373±133 bp) from a previously published ‘adult’ dataset [12]. This composite dataset was assembled into 24,896 contigs (see Table 1; mean contig length = 1068.76±1284.61 nt, longest contig = 20,661 nt, shortest contig = 100 nt); 17,357 of these contigs had a homologue in available transcriptomic data for *C. sinensis* [12], 13,035 or 12,480 in *Fasciola hepatica* or *F. gigantica* [19], [20], respectively, and ∼13,250 had a homologous sequence in genomic data available for *Schistosoma* spp. [17], [21], [22] (Table 2).

**Table 1:** Summary of the ‘old’ (454) and ‘new’ (454+ Illumina) transcriptomes representing *Opisthorchis viverrini*.

	Datasets	
Characterization of transcripts	454	454+ Illumina
Total unique sequences after assembly (100 bp)	55,274	24,896
Largest transcript (bases)	12,042	20,661
Mean transcript length (bases)	484.9	1068.8
Predicted peptides containing a predicted ORF (>50 aa)	49,187	21,026
Mean length of predicted peptides (aa)	165.8	328.9
Maximum length of predicted peptide (aa)	4,014	6,832
Minimum length of predicted peptide (aa)	51	51
Full-length transcripts (containing start and stop codon)	7,266 (13.1%)	4,281 (17.2%)
Partial transcripts with start codon only	12,295 (22.2%)	2,358 (9.4%)
Partial transcripts with stop codon only	14,616 (26.4%)	9,636 (38.7%)
Containing transmembrane domains	3,453 (7.0%)	8,204 (39.0%)
Sequences with signal peptides	3,305 (6.7%)	5,441 (25.9%)
Putative excretory/secretory proteins	1,470 (2.9%)	545 (2.6%)
Number of transcripts unique to transcriptome	24,330	5,417
Number of unique transcripts that could be annotated	817 (3.3%)	1,309 (24.2%)

Homology Searches		
KOBAS		
Number of transcripts with KEGG orthologous terms (unique terms)	-	6,277 (2,823)
InterPro Scan		
Number of transcripts with InterproScan terms (unique terms)	-	9,827 (4,243)
BLASTx		
Number of hits in the NCBI-nr database	-	13,784 (55.4%)

**Table 2:** Comparative transcriptomic analyses between *Opisthorchis viverrini* and other parasitic trematodes [12], [19]–[22].

	*O. viverrini* sequences (n = 24,896) with homology (%)		
Predicted proteins similar to those in:	*E*-value≤1×10^−5^	E-value≤1×10^−15^	E-value≤1×10^−30^
Transcriptomic data			
*Opisthorchis viverrini*	18729 (75.23)	16807 (67.51)	14506 (58.27)
*Clonorchis sinensis*	17357 (69.72)	15227 (61.16)	12964 (52.07)
*Fasciola hepatica*	13035 (52.36)	10693 (42.95)	8219 (33.01)
*Fasciola gigantica*	12480 (50.13)	10233 (41.10)	7782 (31.26)
*Genomic data*			
*Schistosoma mansoni* a	13252 (53.23)	11114 (44.64)	8900 (35.75)
*Schistosoma haematobium* a	13277 (53.33)	11050 (44.38)	8742 (35.11)
*Schistosoma japonicum* a	13277 (53.33)	11049 (44.38)	8734 (35.08)

a Sequence comparisons made against complete gene sets predicted from genome sequence data.

In a direct comparison with the ‘old’ *O. viverrini* transcriptome [12], 18,729 of the ‘new’ contigs had a close homologue (*E*-value cutoff: 1×10^−5^; Table 2). Based on comparative alignment of these homologous contig pairs (i.e., from the ‘old’ and ‘new’ assemblies), we identified 2,688 insertion and 1,311 deletion events associated with a homopolymeric region of ≥4 nt relating to 3,086 distinct transcripts. In total, 313,515 such homopolymers were detected, suggesting a total indel error rate of ∼1.3% in the previous dataset [12]. Correction of these indel errors coincided with the improved ORF lengths in the new assembly (n = 21,026; ≥50 amino acids [aa] in length), which were significantly longer than those achieved using 454 data alone (mean ORF length: 329 *versus* 168 aa, respectively; Table S1) and contained more information to facilitate functional annotation (e.g., 2863 unique PFAM domains in the 454+Illumina data assembly *versus* 2541 such domains in the 454 only dataset; Table S2).

Of the 21,026 contigs inferred to encode a peptide (≥50 aa) in the present assembly, 65.6% had a homologue (*E*-value cutoff: 1×10^−5^) in a eukaryote in the non-redundant protein database, with 9,827 predicted to encode at least one conserved protein domain (mean of 2.2 domains per sequence; Table 1) and, on the basis of these data, 37.1% of the predicted peptides could be assigned GO terms. For a more specific annotation, 6,277 peptides had an orthologous match in the KEGG database relating to 2,823 distinct KEGG orthologues and 249 conserved biological pathways (Table 1). Signal peptides and transmembrane domains were identified in 5,441 and 8,204 proteins of *O. viverrini*, respectively, and a total of 545 was inferred [28] to represent ES proteins (Table 1).

In addition to annotating these transcripts using such generalist resources, we interrogated specialist databases, in order to identify key protein classes, including kinases, transporters and channel proteins, receptors and peptidases, known to have important functional roles and being druggable in many parasitic helminths [39]. Using this approach, we annotated 333 kinases, 2,827 transporter and/or channel molecules, and 699 peptidases (Table S3). Among the peptidases, which are known to play a range of important functional roles in trematodes [40], the cysteine (58% of the peptidases), serine (20%) and metallo- (20%) peptidases predominated (Table S3), with the remaining classes (threonine and aspartate peptidases) being relatively rare. Interestingly, although metallopeptidases were relatively evenly distributed among ∼30 protein families (e.g., M1, M12B and M13), cysteine and serine peptidases were clearly dominated by a small subset of families, including the C1A (‘papain-like’: n = 62 transcripts), C2 (‘calpain-like’: n = 18), C13 (‘legumain-like’: n = 190), C19 (‘ubiquitin-specific’: n = 64), S1A (‘chymotrypsin’: n = 25) and S8A (‘subtilisin-like’: n = 51) peptidases. It is likely that much of this richness relates to alternative splicing of the mRNA. When the transcripts were clustered based on their ‘definitive’ peptidase domains (i.e., the region that allows classification to family), these transcripts were inferred to relate to 9 C1A, 6 C2, 5 C13, 20 C19, 5 S1A and 5 S8A domains based on homology to proteins in the MEROPs database.

**Figure 1:**
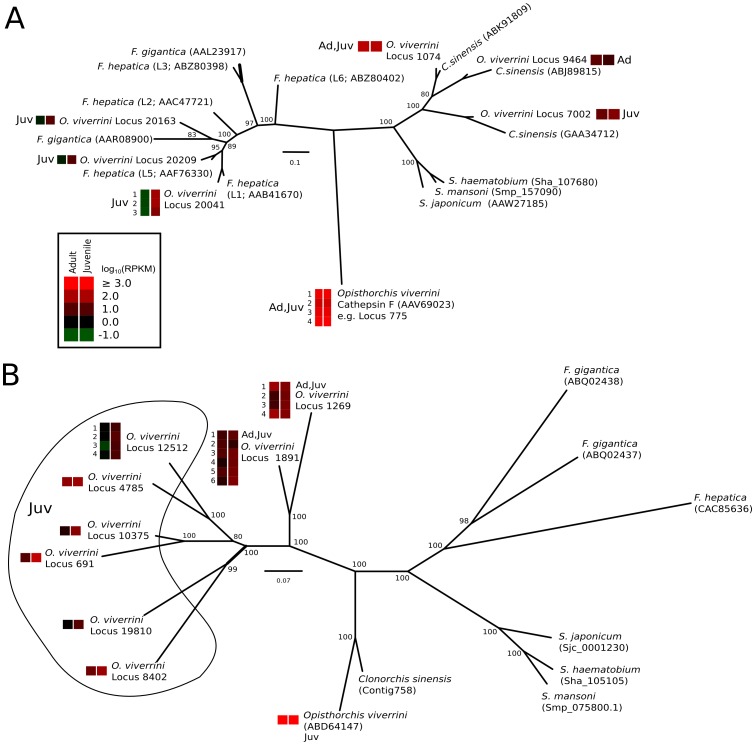
Phylogenetic relationships of key groups of cysteine proteases among selected parasitic trematodes. (A) cathepsin L and (B) asparaginyl endopeptidase (AEP) enzymes. Values indicated on the branches represent Bayesian inference bootstrap support. Opisthorchis viverrini sequences differentially (Ad or Juv) or constitutively (Ad,Juv) transcribed in the adult (Ad) or juvenile (Juv) stage are indicated. Transcription is expressed as log10-transformed reads per kilobase per million reads (RPKM). The GenBank accession number of each sequence is given.

The expansion of the C13 ‘legumain’-like molecules (Pfam code: PF01650) was of interest, considering that only one of them was predicted previously for *O. viverrini* [41] and a single ‘legumain’-like gene has been annotated for each trematode species for which extensive genomic or transcriptomic data are available (see Figure 1), with the exception of *F. gigantica* for which two such genes have been described [42]. In the present dataset, 190 transcripts had high BLASTx homology to 1 of 5 unique C13 peptidase domains in the MEROPs database. Subsequently, we conducted a multi-alignment of these transcripts and identified 9 unique C13-domain sequence types. It is likely that the discrepancy between the alignment and homology data relates to an under-representation of homologous sequences in the MEROPs database rather than a misidentification of the sequences themselves. However, to assess the support for the annotation of the C13 ‘legumains’, we conducted a phylogenetic analysis of a consensus alignment for each of the 9 unique C13-like domain sequences inferred from the *O. viverrini* transcripts as well as sequence data for homologues from all other parasitic trematodes for which extensive datasets are available (i.e., *C. sinensis*, *Fasciola* spp. and *Schistosoma* spp.) (see Figure 1). This analysis showed a clear radiation of the C13-like peptidases in *O. viverrini*, with all of the sequences described here forming a distinct, monophyletic clade. These novel C13 sequences appear to relate most closely to C13s described previously for the Opistorchiidae. The species of Fasciolidae (i.e., *F. hepatica* and *F. gigantica*) and Schistostomatidae (i.e., *S. haematobium*, *S. japonicum* and *S. mansoni*) also formed monophyletic clades by family. Posterior probability (pp) support for each of these major clades was 1.00.

### Differential transcription between adult and juvenile stages of *O. viverrini*

In addition to refining the current assembly of the *O. viverrini* transcriptome, the RNA-Seq data generated here allowed, for the first time, a detailed assessment of quantitative differences in transcription between juvenile and adult stages of this species. Of the 24,896 contigs in the current assembly, 19,283 did not differ significantly in their observed levels of transcription between these two stages (Bonferroni transformed p-value: >0.01; RPKM difference ≤2-fold), whereas 3,020 and 2,593 transcripts were differentially transcribed (p-value≤0.01; RPKM difference ≥2 fold) in juveniles and adults, respectively (Table S3).

A significant percentage (11%) of molecules with increased transcription in juvenile *O. viverrini* encoded peptides associated with energy metabolism, including oxidative phosphorylation (e.g., cytochrome *c* oxidase and NADH dehydrogenase (ubiquinone) 1 alpha sub-complex 1), fatty acid metabolism and amino acid (e.g., valine, leucine, isoleucine and lysine) degradation (see Table S4, S5, S6). In the juvenile stage, increased transcription was observed for both secreted and non-secreted cysteine peptidases (e.g., families C13 and C1A; see Table S3 and S7), including molecules known to be involved in protein catabolism, proteolysis and lysosome-specific pathways in other species of fluke [43]. Of the expanded C13 legumain-like sequences predicted, six had increased transcription in the juvenile stage, as did four unique C1 cathepsin-like (PF01650) cysteine peptidase domains, each of which has close homology to cathepsin L genes in *Clonorchis* and/or *Fasciola*. To support the annotation of these transcripts, as for the C13 legumains, each unique C1 cathepsin-like domain was aligned with cathepsin L-like sequences from *C. sinensis* [44], *Fasciola* spp. [45] and *Schistosoma* spp. [46], and then clustered by Bayesian inference (see Figure 1). In total, we identified six unique C1 domains using this approach. Although three additional, unique domains were identified based on homology with data in the MEROPs database, these transcripts were not sufficiently complete to allow an assessment of the entire C1 domain and thus were not considered further. Based on these analyses, three of the six putative cathepsin L-like domains clustered with the other representatives of the Opistorchiidae (one juvenile-enriched, one constitutively transcribed and one adult-enriched) and three with the recognized cathepsin Ls from *Fasciola* (all juvenile enriched). Nodal support for these clusters was high in all instances (pp values ranging from 0.8 to 1.0).

Significantly increased transcription in the adult stage of *O. viverrini* related to nucleotide processing and oocyte meiosis. For example, GO terms that were highly represented in the adult included nucleoside, nucleobase and nucleotide kinase activity as well as ribonucleotide binding (for adenylate, nucleoside diphosphate and casein kinases) (see Table S4). Transcription linked to nucleotide replication and processing was significantly higher in the adult compared with the juvenile stage (Table S5). In addition, chromosome–specific proteins (e.g., histone-lysine N-methyl transferase and chromosome-transmission fidelity protein), DNA repair and recombination proteins (e.g., meiotic recombination protein and DNA polymerases) as well as proteins involved in the processing of mRNA ( = spliceosome), such as integrator complex subunit (INTS) and splicing factor (e.g., SFRS2), were enriched in adult *O. viverrini* (Table S3 and S6). Pathways associated with the nucleotide synthesis, including purine and pyrimidine metabolism, were also enriched in this developmental stage (Table S4). Furthermore, transcription associated with oocyte meiosis was higher in the adult stage (Table S3 and S7), and included transcripts encoding adenylate cyclases, ribosomal kinases and egg-specific antigens.

## Discussion

*Opisthorchis viverrini* (Trematoda; Platyhelminthes) is a socioeconomically important liver fluke that affects ∼9 million people in southeast Asia and, due to poorly understood mechanisms, is one of a small number of parasitic helminths known to directly cause malignant cancer [5]. In an effort to better understand the biology of this neglected parasite, transcripts from the adult stage were sequenced by 454 technology in a previous study [12], yielding ∼55,000 sequences (≥100 nt) predicted to encode ∼49,000 peptides of ≥50 aa. Although this published dataset [12] provided significant, new insights into the transcriptome of *O. viverrini*, the authors acknowledged that substantial gaps remained to be addressed. Specifically, the published assembly was described as being fragmented and, due to limitations in the sequencing chemistry used, potentially contained homopolymer-associated sequencing errors [12]. In addition, because only the adult stage of *O. viverrini* was represented and the initial study focused on a qualitative exploration of the transcriptome, quantitative assessment of transcription during the life-cycle of this parasite was not possible at the time.

In an effort to overcome these limitations and enhance the transcriptomic data available for this important parasite, we generated ∼25 million 50 bp (single-end) sequence reads using the Illumina platform, allowing transcription levels to be quantitated in the juvenile or adult stages of *O. viverrini*. An enhanced (‘new’) assembly was achieved from the combined output of the Illumina platform (including data for each stage) and the raw 454 data generated in the previous study [12]. Comparative analyses conducted here demonstrate that this approach improved considerably the assembly of the transcriptome. Notably, the number of contigs (≥100 bp) in the ‘new’ combined assembly (n = 24,869) was ∼31,000 fewer than in the previous assembly [12], and both the N50 and N90 assembly metrics increased substantially (by 360 and 225% respectively), with reductions particularly in the number of short sequences (i.e, <200 bp: n = 11,177 and 5,175 in the ‘old’ and ‘new’ assemblies, respectively) (Figure 1). This reduction in the number of sequences appears to relate specifically to an enhanced assembly and a reduction of redundancy overall, with 55.9% of the sequences and 80.4% of all contigs from the 454-only assembly having a close match in the new assembly. Indeed, of the remaining 24,330 ‘454-unique’ sequences (contigs or singletons), only 1,480 encoded peptides of ≥50 aa with homology to proteins in other eukaryotes (excluding likely contaminants, such as fungal or vertebrate sequences) in a functional database (Table S8). Importantly, the mapping of the raw Illumina reads to the ‘454-unique’ sequences indicated little coverage in the present dataset (Table S9), suggesting that the transcripts represented by these sequences were of low abundance in the adult and juvenile stages from which mRNA was purified. Notably, the 454 data were generated from a normalized cDNA library, in order to specifically enrich for such lowly transcribed sequences [12].

A comprehensive analysis of the enhanced dataset indicated that the transition from the juvenile to the adult stage of *O. viverrini* relates primarily to a down-regulation of metabolic pathways (likely in response to the reduced growth demands of the organism) and, predictably, an increase in pathways associated with DNA replication and reproduction. It is likely that the increased transcription of these molecules relates to the production of eggs, sperm and embryos, a hypothesis supported by the increased transcription in adults of key meiosis-related genes such as the meisosi specific serine/threonine kinase *mek1*. These findings are consistent with those reported previously for this developmental transition in other flukes, such as *Schistosoma haematobium* [17]. Many of the genes typically associated with highly specific reproductive functions (i.e., spermatogenesis) appear to be constitutively transcribed in the juvenile and adult stages investigated here. Exceptions to this relate primarily to the transcription of genes expressed in the terminal stages of the reproductive process, such as vitelline B (involved in egg-yolk production) [47] and homologues of the tyrosinase genes *tyr*1 and *tyr*2, known to be associated with late-phase egg shell synthesis in *Schistosoma* spp. [48]. Indeed, these genes were among the most highly transcribed genes in adult *O. viverrini*.

All hamsters used in the present study to produce *O. viverrini* were helminth-free and infected at the same time point, with the juvenile worms being harvested two weeks following inoculation. The pre-patent period for *O. viverrini* is at least four weeks [4], and all specimens yielded at two weeks were confirmed to be immature worms. Our data suggest that the transcription of many of the genes associated with the early phases of reproductive process (e.g., spermatogenesis and oogenesis) begins long before the worm reaches adulthood. However, an alternative hypothesis is that these genes have different functional roles at different stages during the life-cycle of *O. viverrini*.

The enhanced assembly of the transcriptome allowed greater insights into a variety of important and/or druggable groups of molecules, including receptors and transporters, kinases and peptidases. Most notably, we observed a substantial enhancement in the assembly of the complex cysteine peptidase families, including those representing C1 and C13, which are noted for their important functional roles in many helminths [40]. In particular, we assembled 367 transcripts encoding a conserved cysteine peptidase domain based on BLASTx homology, and further characterized particular families of ES molecules based on subsequent phylogenetic analysis of known homologues from other helminths. Based on our analysis, a striking expansion of the C13 legumains in *Opisthorchis* relative to all other parasitic helminths for which extensive genomic and/or transcriptomic data are available was detected. This expansion appears to relate primarily to an independent lineage positioned close to, but, clearly, distinct from the nearest homologous sequence in another helminth species, being the single C13 legumain-like peptidase identified in *C. sinensis*, although we did detect also a close orthologue of this sequence. Interestingly, transcription data relating to these sequences suggest that all but two of the C13 legumain-like sequences detected here for *O. viverrini* were significantly up-regulated in the juvenile relative to the adult stage. The legumains, or asparaginyl endopeptidases (AEPs), are known to cleave peptide bonds on the carboxyl-terminus of asparagine residues [49] and, in *S. mansoni*, have been implicated in the trans-processing and activation of cathepsin B haemoglobinase, thus enabling the degradation/digestion of host haemoglobin [50]–[52]. The asparaginyl endopeptidase sequences characterized here include typical histidine and cysteine residues, essential for enzymatic activity, suggesting that they are indeed functional. The specific divergence and radiation of these peptides in *O. viverrini*, and their transcription in the juvenile stage suggests an essential role during a critical phase of development. A likely hypothesis is that these molecules have radiated in *O. viverrini* to facilitate the exploitation of particular proteins in bile as a novel food source and/or cell detritus resulting from epithelial cell (cholangiocyte) turnover and/or immune cells undergoing diapedesis through the epithelium, particularly in chronically infected animals. The up-regulation of transcripts linked to these proteins is co-incidental with those associated with a variety of proteins involved specifically in metabolic pathways in the juvenile stage, which, at least circumstantially, supports this hypothesis.

Also notable among the cysteine peptidases were the cathepsin L-like proteins. Close homologues of individual sequences defined previously in *C. sinensis* [44] were identified and clustered using phylogenetic inference. However, intriguingly, we also detected specific, close homologues in *O. viverrini* of genuine cathepsin Ls reported for *Fasciola* spp. [43]. These sequences do not segregate into clades by species, rather are positioned on the tree as monophyletic pairs (one *O. viverrini* sequence grouping with one *Fasciola* sequence) based on the nearest homologue in *Fasciola*, suggesting a common evolutionary (i.e., orthologous) origin for each gene, rather than an independent radiation of this group of enzymes, as has been proposed previously for cathepsins of trematodes [43]. Despite this apparent orthologous relationship, we can only speculate as to the specific functional roles of these enzymes in *O. viverrini*. In *Fasciola*, cathepsin Ls appear to be directly involved in tissue penetration (cathepsin L3) and the digestion of host proteins during migration and development of the immature stage (cathepsins L1 and L2) and within the bile duct as adults (cathepsin L5) [43]. To our knowledge, *O. viverrini* does not penetrate host tissues, and, consistent with the understanding of the cathepsin Ls in *Fasciola*, we find no evidence of a homologue of cathepsin L3 in *Fasciola*. We did detect, however, putative orthologues of cathepsins L1 and L5. It is likely that these enzymes are critical also for feeding in *Opisthorchis*. For the *O. viverrini* cathepsin L1 orthologue, this inference is supported by differential transcription data, with the peptide being significantly up-regulated in the juvenile stage (relative to the adult), consistent with the transcriptional profile known for this molecule in *Fasciola* spp. [43]. In contrast, in *Fasciola*, cathepsin L5 has been reported to be up-regulated in adults, with little evidence of transcription in the juvenile stage [43]. However, in *O. viverrini*, the cathepsin L5 orthologue is clearly transcribed at a higher level in the juvenile stage, suggesting a possible difference in the role of this enzyme in the latter species, despite the apparent orthologous origin. Intriguingly, although the *Fasciola*-like cathepsin L sequences appear to be enriched in the juvenile stage, the three *Clonorchis*-like cathepsin Ls, are, generally, represented by a higher level of transcription, and two of them are specifically enriched in the adult stage. Unlike *Fasciola*, for which juvenile stages migrate through the liver parenchyma, both the juvenile and adult stages of *O. viverrini* live in the bile ducts of the host. Therefore, it is possible that *O. viverrini* has developed ‘stage-enriched’ suites of cathepsins that allow both of these stages to fill complementary, but non-overlapping niches within the host (e.g., exploiting similar but distinct food-sources), thus reducing inter-generational competition for resources/nutrients. Clearly, an in-depth exploration of the cathepsin Ls in *O. viverrini* would enable better insights into the feeding mechanisms of these parasites. Our proposals could be explored at a functional level, either through gene knock-down and knockout approaches, which are already established for some parasitic flukes, including *O. viverrini* [53], [54] or through the use of free-living ‘model’ flukes, such as *Schmidtea mediterranea* or *Macrostomum ligano*, in the same way that *C. elegans* has been employed as a (surrogate) functional tool for parasitic nematodes [11]. Given the utility of these model flukes as tools to study tissue and nerve regeneration [55]–[57], ongoing efforts to sequence their genomes and transcriptomes (of different developmental stages) should assist in the establishment of effective functional genomic tools for trematodes [58]. The development of such tools would provide major support toward the development of novel interventions against socioeconomically important trematodiases.

Through the use of Illumina-based sequencing, the present study provides a deep insight into the battery of cysteine peptidases that *O. viverrini* can deploy [43], specifically in relation to the radiation of the C13 legumains and the presence of cathepsin Ls orthologous to those of *Fasciola* spp. These findings contrast the existing hypothesis for the evolution of the peptidases in flukes (i.e., family-specific radiation/evolution) [43]. The finding that the cathepsin Ls of *O. viverrini* do not appear to have an homologue in *C. sinensis*, despite the close biological relationship shared by these species (both being members of the Opistorchiidae), suggests that radiation prior to differentiation of the major trematode families, followed by subsequent gene loss, may also have shaped the cathepsins in trematodes [43]. This interpretation is further supported by the presence of cathepsin F-like proteins encoded in the adult transcriptome of *F. hepatica* [19]. Notably, previous knowledge and understanding of these molecules in trematodes has largely been based largely on proteomic observations, which tend to be biased toward abundantly expressed proteins [59]. Indeed, taking into account only the highly transcribed sequences in the current dataset, the results of the present study are consistent with the existing hypotheses for opistorchiids (i.e., a reliance on cathepsin Fs, proliferation of cathespin Bs and a single C13 legumain-like homologue) and for the family specific radiation of these enzymes [43]. However, it is well established that one of the strengths of RNA-Seq technology is its ability to resolve both lowly transcribed sequences and alternative splicing events which are often not detectable using traditional technologies (e.g., microarray) or even sensitive proteomic tools [60]. This point is clearly worthy of note, given that RNA-Seq has not be widely deployed for the characterization of other socioeconomically important flukes (e.g., species of *Schistosoma*, *Fasciola* and *Clonorchis*). It may well be the case that similar expansions of the cysteine peptidases and specialization relating to the exploitation of specific food sources has occurred in a range of fluke species, but has, as yet, not been resolved due to the limitations of previous technologies. Clearly, given the key functional roles that many of these molecules play in fluke biology and pathogenesis [40], including in *O. viverrini* [61], [62], deeper exploration of fluke cathepsins using RNA-seq technology is needed. Coupling such investigations with expanded genomic sequencing of key parasites would provide much greater insight into the role that alternative splicing may play in the transcriptional biology of flukes; an area which to date has been explored only to a limited extent.

## Supporting Information

Table S1 **Summary of the identification and correction of homopolymer errors in the 454-only and 454+Illumina assemblies of the transcriptome of** ***Opistorchis viverrini*** **.**
(XLSX)

Table S2 **Comparative assessment of the number of unique PFAM domains per assembled transcript between the ‘old’ (i.e., 454-only) and ‘new’ (i.e., 454+ Illumina) transcriptome of** ***Opistorchis viverrini*** **.**
(XLSX)

Table S3 **Information on the annotation of each transcript assembled using the combined 454 and Illumina data and assessment of their relative levels of transcription in the juvenile and adult stages of** ***Opistorchis viverrini*** **.**
(XLSX)

Table S4 **Comparative summary of the enriched transcription of conserved biological pathways in adult or juveniles of** ***Opistorchis viverrini*** **based on orthology to molecules in the Kyoto Encyclopaedia of Genes and Genomes (KEGG).**
(XLSX)

Table S5 **Comparative summary of the functional classes of adult or juvenile-enriched transcripts of** ***Opistorchis viverrini*** **based on the classification of their encoded protein domains in the Gene Ontology (GO) based classification system (** http://www.geneontology.org/ **).**
(XLSX)

Table S6 **Comparative summary of the functional classes of adult or juvenile-enriched transcripts of** ***Opistorchis viverrini*** **based on the presence of conserved protein domains annotated in the PFAM database (** http://pfam.sanger.ac.uk/ **).**
(XLSX)

Table S7 **Summary of the peptidases characterized in the** ***Opistorchis viverrini*** **transcriptome based on homology to proteins in the MEROPS database (** http://merops.sanger.ac.uk/ **) and information on their levels of transcription in juvenile and adult stages of this parasite.**
(XLSX)

Table S8 **Summary of annotation information available for transcripts present in the 454-only assembly but with no homologue in the 454+Illumina assemble of the** ***Opistorchis viverrini*** **transcriptome.**
(XLSX)

Table S9 **Summary of the number of 454 and Illumina reads respectively mapping to each transcript in the 454-only and 454+Illumina transcriptome assemblies for** ***Opistorchis viverrini*** **.**
(XLSX)
